# Full complex amplitude control of second-harmonic generation via electrically tunable intersubband polaritonic metasurfaces

**DOI:** 10.1126/sciadv.adw8852

**Published:** 2025-07-25

**Authors:** Jaeyeon Yu, Jaesung Kim, Hyeongju Chung, Jeongwoo Son, Gerhard Boehm, Mikhail A. Belkin, Jongwon Lee

**Affiliations:** ^1^ Department of Electrical Engineering, Ulsan National Institute of Science and Technology (UNIST), Ulsan 44919, Republic of Korea.; ^2^ Center of Integrated Nanotechnologies, Sandia National Laboratory, Albuquerque, NM 87185, USA.; ^3^ Walter Schottky Institute, Technical University of Munich, Am Coulombwall 4, Garching 85748, Germany.

## Abstract

Nonlinear intersubband polaritonic metasurfaces based on coupling of the intersubband nonlinear optical response of quantum-engineered semiconductor heterostructures and electromagnetic modes of nanoresonators provide efficient frequency mixing with moderate pump intensities. The resonant nonlinear optical response, represented as a complex function, can be modulated via Stark tuning of intersubband transition energies under applied voltages. However, achieving full complex amplitude control (both phase and magnitude) remains challenging. In this work, we present and experimentally validate electrically tunable nonlinear intersubband polaritonic metasurfaces that achieve complete complex amplitude control for second-harmonic generation (SHG). Through a design featuring two in-plane flipped meta-atoms per unit cell, we achieve complete electrical control of both the amplitude and phase of the metasurface second-order nonlinear susceptibility, with a tuning range of 0 to 30 nm V^−1^ for the magnitude and 0-2π for the phase of the nonlinear optical response. Using these properties, we achieve complete on-off SHG modulation and beam diffraction tuning through electrically controlled amplitude and phase gratings.

## Teaser

Electrically tunable metasurfaces enable complete control of the complex amplitude of SHG signals at the subwavelength scale.

## INTRODUCTION

Nonlinear optical metasurfaces, consisting of two-dimensional arrays of subwavelength-scale artificial structures with strong nonlinear optical responses, offer a number of advantages over bulk nonlinear crystals. These include relaxed phase-matching constraints and ability to control of nonlinear responses at a deeply subwavelength scales, which enables manipulation of wavefront and polarization state of the output beam (*1*–*3*). Based on these properties, innovative applications such as new frequency generation for light sources (*1*–*3*), holography and image encoding (*4*–*9*), nonlinear optical switching and modulation (*10*–*15*), and quantum light sources with possibility of generating multifrequency quantum states, including cluster states (*16*–*20*), were demonstrated. To further exploit the potential of nonlinear optical metasurfaces, it is crucial to achieve complete electrical control over the complex amplitude (both in magnitude and in phase) of nonlinear optical response at a level of an individual metasurface unit cell. Recently, research on electrically tunable second- and third-harmonic generation (SHG and THG) using intersubband polaritonic metasurfaces has demonstrated the potential of electrical control of the nonlinear optical response in these structures (*11*, *15*). Intersubband polaritonic metasurfaces are hybrid structures that couple intersubband transitions (ISTs) between confined electron states in n-doped semiconductor heterostructures with electromagnetic modes of subwavelength resonators (*21*, *22*). It has been shown that these metasurfaces can produce second- or third-order nonlinear responses four to eight orders of magnitude larger than that in bulk nonlinear crystals (*23*, *24*), enabling efficient SHG and THG in deeply subwavelength films using only moderate pump intensities on the order of few tens of kW cm^−2^ (*25*–*27*).

The resonant intersubband nonlinearities exhibit the strongest nonlinear response when the input pump frequency is resonant with ISTs, and both the amplitude and phase of the nonlinear optical response exhibit substantial changes near the resonant frequency. In previous studies, we demonstrated that the complex amplitude of the intersubband nonlinear susceptibility could be modulated by an applied voltage using spatially separated electron subbands in a heterostructure (*11*, *15*). Furthermore, by combining the heterostructure with plasmonic cavity structures, we experimentally demonstrated electrical control over the spectrum, intensity, and phase of SHG or THG at the individual level of a subwavelength-scale meta-atoms. However, in these earlier efforts, we were unable to fully decouple the magnitude and phase of the nonlinear response. Moreover, the phase tuning range was limited to the level of a π phase, thereby restricting the potential for wavefront tuning of the nonlinear output.

**Fig. 1.**
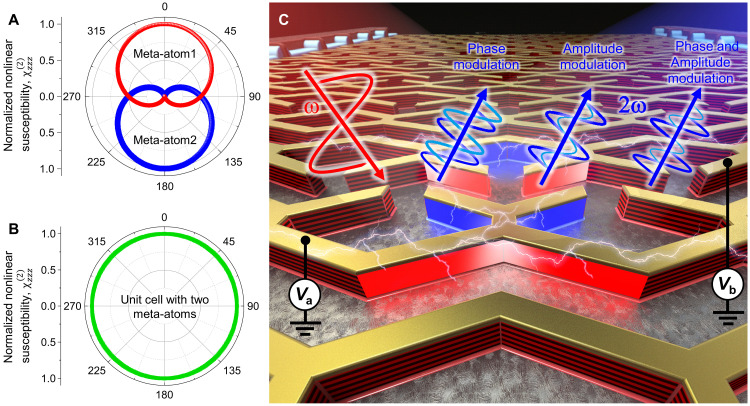
Electrical complex amplitude control of SHG using nonlinear polaritonic metasurface. (**A**) Normalized nonlinear susceptibilities represented in a polar plot, following a cardioid function (red: meta-atom 1 exhibiting an ordinary cardioid function; blue: meta-atom 2 exhibiting a cardioid function with π-phase shift). (**B**) Normalized nonlinear susceptibility resulting from the in-phase summation of the two cardioid functions in (A). (**C**) Conceptual illustration of complex-amplitude-controllable nonlinear polaritonic metasurface. Red and blue arrows represent input pump beam at the fundamental frequency (FF) ω and second-harmonic frequency 2ω output signal, respectively. With the two applied voltages, *V*_a_ and *V*_b_ to the two meta-atoms in a unit cell structure, phase-only modulation, amplitude-only modulation, and both phase and amplitude modulation of second-harmonic signal are possible.

Here, we propose and experimentally demonstrate electrically tunable nonlinear intersubband polaritonic metasurfaces that allow complete control over both the magnitude and phase of the SHG signal. To that end, we quantum-engineer our intersubband heterostructure to have ISTs for doubly resonant SHG at λ ≈ 9.8 μm at zero bias voltage and to have exactly the same Stark tuning coefficients for one to two and two to three electron states transition energies. In this case, the parametric dependence of the real and imaginary parts of the normalized intersubband nonlinear susceptibility [ $\chi_{\text{norm}}^{\left(2\right)}$ ] on bias voltage can be mathematically shown (see Text S1) to follow a form similar to that of a Cardioid function0 { $r\left(\theta \right)=\frac{1}{2}\cdot \left[1-\text{cos}\left(\theta \right)\right]$ } in the complex plane, as illustrated in Fig. 1A. Our metasurface is designed with a unit cell consisting of two meta-atoms that are flipped in-plane relative to one another. In this case, the effective nonlinear response of the metasurface unit cell is a sum of two Cardioid functions with a π phase shift $\left\{\;r\left(\theta \right)\right.$ and $\left.r^{\prime }\left(\theta^{\prime }\right)=\frac{1}{2}\left[1-\text{cos}\left(\theta^{\prime }-\pi \right)\right]\right\}$ , which mathematically forms a circle in the complex plane at the same phase ( $\theta =\theta^{\prime }$ ) (see Fig. 1B) (*28*). By applying separate bias voltages to each of the two meta-atoms in a unit cell, similar to the case where $\theta^{\prime }\neq \theta$ , we achieve independent modulation of both the amplitude and phase of the effective second-order nonlinear susceptibility of the metasurface, with experimentally measured values of 0 to 30 nm V^−1^ for the magnitude and 0-2π for the phase.

## RESULTS

### Operating principles and metasurface design

The concept of our electrically tunable nonlinear metasurfaces is illustrated in Fig. 1C. The metasurface consists of two X-shaped meta-atoms, which are connected to adjacent structures in the lateral direction (*x* direction here). Each row of the meta-atom array is connected to electrodes that allow for the application of two different voltages, *V*_a_ and *V*_b_. In this configuration, the amplitude and phase of the second-harmonic (SH) signal generated from the metasurface under a fundamental frequency (FF) pump beam can be controlled at the unit cell level by adjusting two applied voltages. This allows for modulation of SHG intensity and wavefront phase control.

**Fig. 2.**
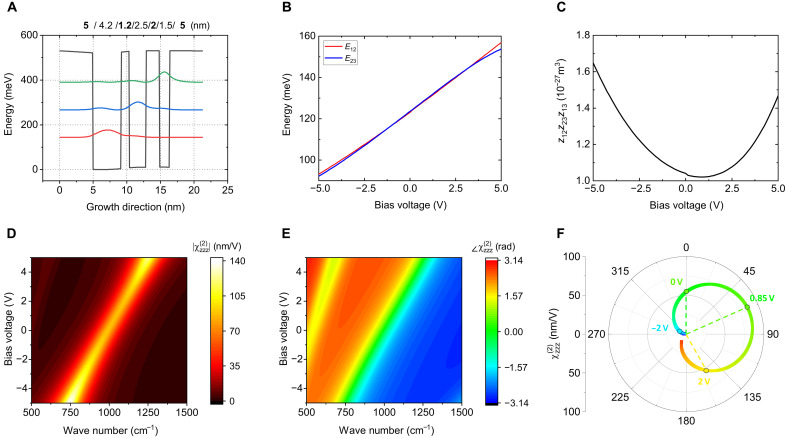
Optical properties of MQWs. (**A**) Conduction band structure of one period of three coupled quantum wells (black solid line) and the probability densities of the confined electron states (*E*_1_: red, *E*_2_: blue, and *E*_3_: green solid curve). The thicknesses of the In_0.53_Ga_0.47_As (well) and Al_0.48_In_0.52_As (barrier) are indicated with thin and bold numbers above the graph, respectively. (**B**) Changes in the IST energy (*E*_12_: red and *E*_23_: blue) with applied voltage. (**C**) Absolute value of the product of the dipole matrix elements, *Z*_21_*Z*_32_*Z*_31_, as a function of voltage. The minimum value and minimal variation occur at 0.85 V. (**D** and **E**) Changes in the (D) magnitude and (E) phase of the second-order susceptibility of the MQW with applied voltages. (**F**) $\chi_{\mathrm{zzz}}^{\left(2\right)}$ at the central wavelength of 9.8 μm converted into polar coordinates over the voltage range of −5 to 5 V. The last point in blue corresponds to −5 V, the last point in red corresponds to 5 V, and the point in green corresponds to 0 V. The color gradient changes with voltage, and specific points at −2, 0, 0.85, and 2 V are marked on the graph.

A coupled three quantum well unit structure was designed using an In_0.53_Ga_0.47_As/Al_0.48_In_0.52_As heterostructure, as shown in Fig. 2A, where three spatially separated electron subbands were induced. The layer sequence of the multiple quantum well (MQW) structure is **5**/4.2/**1.2**/2.5/**2.5**/1.5/**5** nm, where the boldface indicates Al_0.48_In_0.52_As barriers, and the first 4.2-nm well is n-doped with a density of 3.5 × 10^18^ cm^−3^. In this study, a 500-nm-thick MQW layer, consisting of 23 repetitions of this unit structure, was used. The giant second-order nonlinear susceptibility $\chi_{\mathrm{zzz}}^{\left(2\right)}$ is induced by resonant ISTs between the three electron subbands confined within the MQW as shown in Fig. 2A (*z* is the growth direction). The value of $\chi_{\mathrm{zzz}}^{\left(2\right)}$ is expressed as a function of the pump frequency ω and applied bias voltage *V* as follows (*23*)

$$\begin{array}{c} \chi_{\mathrm{zzz}}^{\left(2\right)}\left(\omega \to 2\omega ,V\right)\\ \approx \frac{N_{e}e^{3}}{\varepsilon_{0}}\frac{Z_{12}\left(\;V\right)Z_{23}\left(\;V\right)Z_{31}\left(\;V\right)}{\left[\hbar \omega -E_{21}\left(\;V\right)\left.-i\hbar \gamma_{21}\right]\left[2\hbar \omega -E_{31}\left(\;V\right)-i\hbar \gamma_{31}\right]\right.} \end{array}$$ (1)

where *N*_e_ is the averaged electron density, *e* is the electron charge, ω is the pump frequency, ℏ is the reduced Planck constant, *E*_ij_(*V*) and *eZ*_ij_(*V*) are the IST energy and dipole moment as a function of bias voltage *V*, and $\hbar \gamma_{\mathrm{ij}}$ is the half-width at half-maximum linewidth of the IST between electron subband *i* and *j*. It can be observed that $\chi_{\mathrm{zzz}}^{\left(2\right)}\left(\;V\right)$ exhibits a complex amplitude due to the presence of two resonant denominators in Eq. 1, and the IST energies, *E*_12_ and *E*_13_ (or *E*_23_), can be tuned by the applied voltage due to the quantum-confined Stark effect of the ISTs. The metasurface is further designed so that the voltage dependent changes of transition energies Δ*E*_21_(*V*) and Δ*E*_32_(*V*) are identical. This characteristic is achieved through controlling the spatial separation of electron subbands 1 to 3 (see text S2). The optimization of the MQW design was carried out using a Poisson-Schrodinger solver, and the IST energies, *E*_21_(*V*) and *E*_32_(*V*), and the product of the three transition dipole elements $Z_{12}\left(\;V\right)Z_{23}\left(\;V\right)Z_{31}\left(\;V\right)$ , are shown in Fig. 2 (B and C, respectively). For the IST energies *E*_21_ and *E*_32_, it was confirmed that both values exhibit nearly identical values and trends as the applied voltage increases. The product of the three transition dipole elements reaches a minimum near 0 V and tends to increase as the applied voltage rises. Figure 2D shows the magnitude of the second-order susceptibility, $\chi_{\mathrm{zzz}}^{\left(2\right)}\left(\;V\right)$ , as a function of voltage, calculated using Eq. 1. As the IST energies shift, the spectral peak of $|\chi_{\mathrm{zzz}}^{\left(2\right)}\left(\;V\right)|$ blue-shifts under positive voltage and red-shifts under negative voltage. Because $\chi_{\mathrm{zzz}}^{\left(2\right)}\left(\;V\right)$ is a complex function, its phase also varies with voltage, as shown in Fig. 2E. The voltage-dependent magnitude and the phase of $\chi_{\mathrm{zzz}}^{\left(2\right)}\left(\;V\right)$ , calculated at the central wavelength of *E*_12_ = 126.5 meV (9.8 μm), are plotted on the complex plane in Fig. 2F. The changes in magnitude and phase with applied voltage from −5 to +5 V for the 500-nm-thick MQW layer follow the shape of a cardioid function as expected mathematically (see text S1). If two second-order nonlinear susceptibilities, induced in the same MQWs, follow cardioid functions with a π-phase difference, then their in phase sum will have a constant value (a circle in the polar plot) regardless of the polar angle (*28*). By individually controlling the two phases using applied voltage, it is also possible to access all points within the circle. Therefore, this characteristic allows for the manipulation of the complex amplitude of the nonlinear response as desired.

We note that the “ideal case” of voltage-controlled considered in the Supplementary Materials assumes no variation in the value of the product of dipole moments $Z_{12}\left(V\right)Z_{23}\left(V\right)Z_{31}\left(V\right)$ in Eq. 1 as a function of bias voltage. Some variations in the value of this product are difficult to completely suppress experimentally. As a result, the shape of the $\chi_{\mathrm{zzz}}^{\left(2\right)}\left(V\right)$ curve in the complex plane deviates slightly from the cardioid function shape as indicated in Fig. 2F. Nevertheless, as discussed in the following, this does not prevent us to achieve our goal of a complete control of phase and amplitude of the nonlinear optical response of a metasurface unit cell.

**Fig. 3.**
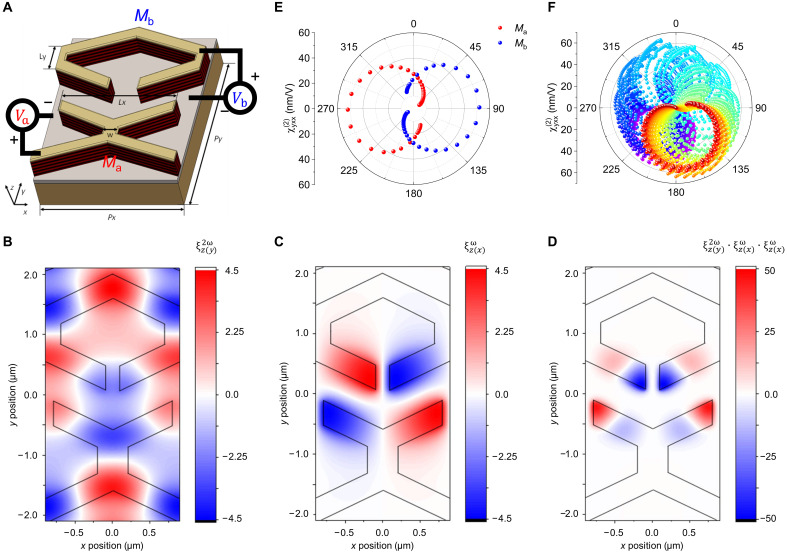
Metasurface design and effective second order susceptibility. (**A**) The unit cell structure of the metasurface. The dimensions are *P_x_* = 1.84 μm, *P_y_* = 4.5 μm, *L_x_* = 1.62 μm, *L_y_* = 0.38 μm, *W* = 0.4 μm, and angle of arm φ = 128°. Meta-atoms are connected along the *x* axis to adjacent meta-atoms. (**B** and **C**) *E_z_* field enhancement of the plasmonic mode induced within the MQWs by (B) *y*-polarized SH incident light and (C) *x*-polarized FF incident light. (**D**) Modal overlap of the *E_z_* fields induced in (B) and (C). In (B) to (D), the *x*-*y* plane is 250 nm below the top metal layer, with the boundary between the MQWs layer and air indicated by a black solid line to represent the volume integration range. (**E**) Effective second-order susceptibilities [ $\chi_{\mathrm{yxx}}^{\left(2\right)\text{eff}}$ ] from meta-atoms, *M*_a_ and *M*_b_, shown in red and blue, respectively. (**F**) $\chi_{\mathrm{yxx}}^{\left(2\right)\text{eff}}$ induced by every combination of voltages *V*_a_ and *V*_b_ within the range of −5 to 5 V at intervals of 0.125 V

Figure 3A shows the unit cell structure of the metasurface designed to control the complex amplitude of the SHG signal. To achieve two second-order nonlinear susceptibilities with a π-phase difference, metasurfaces with two meta-atoms in one unit cell were designed using finite-difference time-domain (FDTD) simulation. Both meta-atoms have the same X-shaped plasmonic nanocavity, with the lower meta-atom (*M*_a_) being rotated 180° in-plane from the upper meta-atom (*M*_b_) and shifted by *P_x_*/2 along the *x* axis. The X-shaped meta-atom has C_1_ rotational symmetry, and the SHG signals generated from the two meta-atoms exhibit a π-phase difference when no voltage is applied. The dimensions of the meta-atom were optimized for an FF wavelength of 9.8 μm and an SH wavelength of 4.9 μm; the specific values are provided in the caption of Fig. 3A. Figure 3 (B and C) shows the *E_z_* field enhancement distribution in the MQW layer for the *y*-polarized light at the SH wavelength of 4.9 μm and *x*-polarized light at the FF wavelength of 9.8 μm, respectively. The *L_x_* of the meta-atom determines the plasmonic resonant wavelength for the *x*-polarized input light at the FF, while the angle of arm φ and dimension *L_y_* determine the resonant wavelength for the *y*-polarized light at the SH wavelength. The effective second-order nonlinear susceptibility element of the nonlinear polaritonic metasurface is calculated using the following equation (*11*, *21*)

$$\begin{array}{c} \chi_{\mathrm{ijk}}^{\left(2\right)\text{eff}}\left(V\right)\\ =\chi_{\mathrm{zzz}}^{\left(2\right)}\left(V\right)\left[\int_{v_{\text{MQW}}}\xi_{z\left(i\right)}^{2\omega }\left(x,y,z\right)\xi_{z\left(j\right)}^{\omega }(x,y,z)\xi_{z\left(k\right)}^{\omega }(x,y,z)\mathrm{dv}\right]/v_{\text{unit}} \end{array}$$ (2)

where *V* is the applied voltage to the MQW structure, $\xi_{z\left(i\right)}^{\omega \;\text{or}\;2\omega }=$$E_{z,\text{MQW}}^{\omega \;\text{or}\;2\omega }/E_{i,\text{inc}}^{\omega \;\text{or}\;2\omega }$ is the enhancement factor of the *E_z_* field in the MQW region ( $E_{z}^{\omega }$ or $E_{z}^{2\omega }$ ) normalized to the incident electric field polarized in the *i* direction (*x* or *y*) at the FF ω or the second-harmonic frequency 2ω, *v*_unit_ is the unit cell volume, and *v*_MQW_ is the MQW layer volume in the metasurface unit cell. The volume integral in the square brackets in Eq. 2 is referred to as the modal overlap integral. The highest effective nonlinear susceptibility of our metasurface is produced for *yxx* polarization combination, where the first letter refers to the SH polarization and the last two letters refer to the FF input pump polarization. Figure 3D shows the modal overlap component in the MQW layer for the *x*-polarized FF light and *y*-polarized SH light. Because the plasmonic modes induced in *M*_a_ and *M*_b_ by *y*-polarized SH light have opposite signs, the effective second-order susceptibilities [ $\chi_{\mathrm{yxx}}^{\left(2\right)\text{eff}}$ ] of the two meta-atoms in a unit cell exhibit a π-phase difference. The integral value of the modal overlap over the entire unit structure volume is zero, but the volume integral value of the modal overlap induced in each meta-atom is 0.76. Figure 3E shows $\chi_{\mathrm{yxx}}^{\left(2\right)\text{eff}}$ for each meta-atom (*M*_a_ and *M*_b_) under 9.8-μm incident light, displayed on a polar plot with red and blue dots, respectively. The effective second-order susceptibilities are plotted for voltages ranging from −5 to +5 V in 0.25-V intervals, shifting clockwise as the voltage increases. The combination of these two cardioid functions enables all points within a radius of 55 nm V^−1^ to be represented through a combination of the two voltages. Figure 3F illustrates all possible combinations of voltages *V*_a_ and *V*_b_ from −5 to +5 V at 0.25-V intervals on a polar plot. To enhance clarity, the combinations are color-coded based on *V*_a_, with red indicating combinations where *V*_a_ is −5 V, blue for *V*_a_ at +5 V, and gradient color transitions in between.

### Experimental characterization

**Fig. 4.**
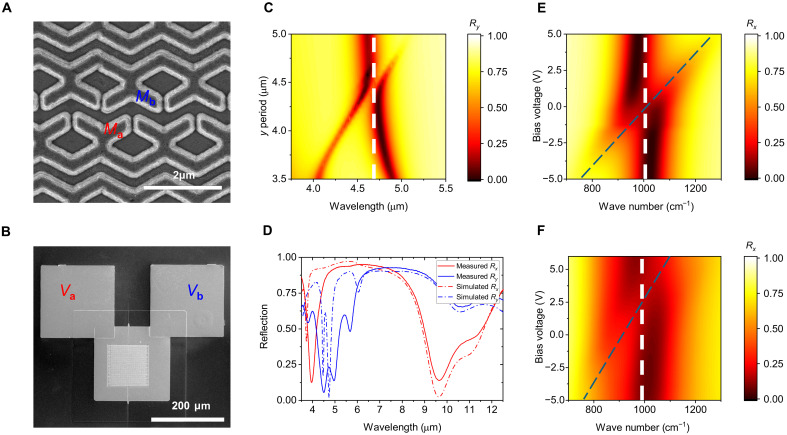
Linear characterization. (**A**) SEM image of the metasurface device. Meta-atoms *M*_a_ and *M*_b_ are electrically isolated by dry etching. (**B**) Full view of the fabricated metasurface device. Electrode *V*_a_ is connected to *M*_a_, and electrode *V*_b_ is connected to *M*_b_. Scales are provided at the bottom right of each image. (**C**) Reflection spectra near the SH wavelength for *y*-polarized light as a function of the *y*-axis unit period *P_y_*. The plasmonic mode for *y*-polarized incident light at a central wavelength of 4.7 μm is indicated by the white dashed line. (**D**) Simulated and measured reflection spectra as a function of polarization at 0 V. (**E**) Simulated reflection spectra near the FF wavelength for *x*-polarized light as a function of voltage. (**F**) Measured reflection spectra near the FF wavelength for *x*-polarized light as a function of voltage. The same voltage was applied to both electrodes, *V*_a_ and *V*_b_. The plasmonic mode for *x*-polarized incident light at a central wavelength of 10 μm and the IST as a function of voltage are indicated by white and blue dashed lines, respectively.

For experimental demonstration, the designed MQW structure was grown using molecular-beam epitaxy, and experimentally measured IST energies were in good agreement with the calculation (see text S3). We fabricated metasurfaces with a 120 μm by 120 μm two-dimensional array of unit cells, following the nano-fabrication process outlined in Materials and Methods and fig. S3. Figure 4A shows a scanning electron microscope (SEM) image of the fabricated metasurface. The dark layer represents the metal layer, while the bright layer represents the MQW layer. Figure 4B shows a low-magnification SEM image of the metasurface device along with the contact pads. *M*_a_ is electrically connected to the electrode *V*_a_ on the left, while *M*_b_ is connected to the electrode *V*_b_ on the right. Figure 4C presents the simulated reflection spectra near the SH wavelength as a function of the *y*-axis period, *P_y_*. The meta-atom exhibits a plasmonic mode for *y*-polarized incident light at a wavelength of 4.7 μm. To reduce diffraction effects caused by SH signals with different phases generated by the two meta-atoms and to efficiently capture the integrated SH signal, the *y*-axis period, *P_y_*, was varied to couple the nonlocal lattice resonance mode with the plasmonic mode. The peak splitting due to this mode coupling can be observed in Fig. 4C. The *P_y_* used in this study is 4.5 μm, resulting in strong resonance peaks at 4.6 and 4.8 μm due to the coupling of the nonlocal lattice resonance mode and the plasmonic mode (*29*–*31*). Figure 4D shows the simulated (dotted lines) and measured (solid lines) reflection spectra of the metasurface for *x*- (red) and *y*- (blue) polarized light at normal incidence. The metasurface design was optimized to provide a strong SHG response for *x*-polarized input at a wavelength of 9.8 μm. Therefore, for *x*-polarized light, a reflection dip was observed near 9.8 μm, and for *y*-polarized light, a sharp reflection dip occurred near 4.9 μm, allowing efficient out-coupling of the SHG into free space. Figure 4E shows the change in simulated reflection spectra for *x*-polarized input as a function of voltage. Reflection dip splitting due to plasmonic-IST coupling is visible, and the asymptotes of this splitting were calculated to estimate the IST absorption peak center as a function of voltage. Figure 4F presents the reflection spectra for *x*-polarized input as a function of voltage for the fabricated metasurface device. The calculated asymptotes of the reflection dip splitting indicate that the IST energy varied from 97.9 to 131.6 meV within the voltage range of −5 to 5 V, with a slope of 3.37 meV V^−1^. This result suggests that the effective voltage applied to the MQWs in the simulation is approximately half of the actual applied voltage.

**Fig. 5.**
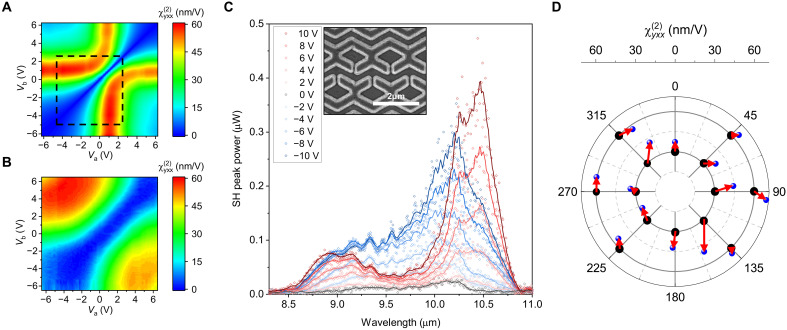
Nonlinear characterization. (**A**) Simulated second-order susceptibility as a function of bias voltage set (*V*_a_ and *V*_b_). (**B**) Second-order susceptibility values converted from the measured SH signal. (**C**) Measured SH peak power spectra (dot denotes measured data, and line denotes moving average) of a full meta-atom structured metasurface as a function of the input pump wavelength for different bias voltages (*V*_a_ only) from −10 to 10 V. (**D**) Magnitude and phase of the second-order susceptibility measured for specific combinations of *V*_a_ and *V*_b_ at 14 points using a nonlinear optical interferogram. The measured points (blue dots) and their corresponding target points (black dots) are connected by red arrows.

We conducted SHG signal measurements using two bias voltages. Figure 5A shows the simulated magnitude of $\chi_{\mathrm{yxx}}^{\left(2\right)\text{eff}}\left(V_{a},V_{b}\right)$ as a function of the voltages *V*_a_ and *V*_b_ for an input wavelength of 9.8 μm, while Fig. 5B represents the experimental $\chi_{\mathrm{yxx}}^{\left(2\right)\text{eff}}\left(V_{a},V_{b}\right)$ extracted from the SHG signal measurements within the voltage range of −6.5 to 6.5 V. Because of additional contact resistance, minor deviations of the experimental IST energies from the design values, and fabrication errors, the measurement results in the −6.5- to 6.5-V range correspond to the −4.8 to 2.4 V results of the simulation shown within the black dashed box in Fig. 5A. When the two voltages are equal (*V*_a_ = *V*_b_), the effective second-order susceptibilities from the two meta-atoms cancel each other, resulting in no SHG signal. However, as the difference between the two voltages increases, the cancellation of the two SHG signals decreases, showing that the effective second-order susceptibility element increases.

To further analyze the nonlinear characteristics of the metasurface, we measured the SHG spectra as a function of the applied voltage for the metasurface. Measured current-voltage relation (*I*-*V*) characterization of the metasurface is provided in fig. S4. Figure 5C presents the SHG signal spectra measured for the metasurface designed for complex amplitude control, with only the *V*_a_ voltage varied from −10 to 10 V during the measurement. The result shows that the metasurface, which contains two meta-atoms generating SHG signals with a phase difference of π, hile the input pump beam is irradiated, exhibits almost perfect signal suppression due to the cancellation of the two SHG signals in the far field. As voltage is applied only to the *M*_a_ of the metasurface, the phase difference between the SHG signals generated by the two meta-atoms deviates from π, and the far-field output signal gradually increases. This characteristic suggests that theoretically, 100% of SHG signal modulation depth or perfect SHG signal on/off switching may be achieved by varying the applied voltage. The measurement result shows that the modulation depth of SHG signal nearly 100% was achieved in the broadband wavelength range of 8.7 to 10.8 μm. It is noted that asymmetric spectral response for the positive and negative bias voltages arises from the slight deviation of the $\chi_{\mathrm{yxx}}^{\left(2\right)\text{eff}}\left(V\right)$ curve in the complex plane from the ideal cardioid function shape, depending on the wavelength. The control sample, which includes only a single meta-atom in the unit cell, does not exhibit this behavior, as shown in fig. S5.

The simulated second-order susceptibility within the voltage range of −5 to +5 V, as shown in Fig. 3F, indicates that all amplitudes and phases within a maximum radius of 55 nm V^−1^ can be represented as a combination of *V*_a_ and *V*_b_. However, within the effective voltage range of −4.8 to 2.4 V shown in Fig. 5A (corresponding to the actual measurement range of −6.5 to +6.5 V as shown in Fig. 5B), there are unavoidable gaps between 315° and 45° and 135° and 225°, preventing complete circular coverage. Nonetheless, within a radius of ~30 nm V^−1^, a full 360° phase coverage can be achieved. To experimentally verify this, we identified the expected voltage combinations of *V*_a_ and *V*_b_ for six points with a magnitude of 60 nm V^−1^ and phase differences of 45°, 90°, 135°, 225°, 270°, and 315° and for eight points with a magnitude of 30 nm V^−1^ and phase differences of 0°, 45°, 90°, 130°, 180°, 225°, 270°, and 315°. We measured the phase changes using an SH interferometer (see text S4). The designed SH optical interferometer ensures accurate phase difference measurement by utilizing the SH beams generated from two fabricated SHG metasurface samples (reference and target). The reference sample and the objective lens were equipped with a linear actuator to vary the optical path length, while probes were used to apply bias voltages to the sample. Fig. 5D presents the measured magnitude and phase differences plotted on a polar plot. Within the voltage range, we were able to fill the interior of the circle representing a 360° phase coverage with a radius of 30 nm V^−1^. With higher voltages, complete phase coverage across larger radii could be achieved, enhancing the system’s tunability.

**Fig. 6.**
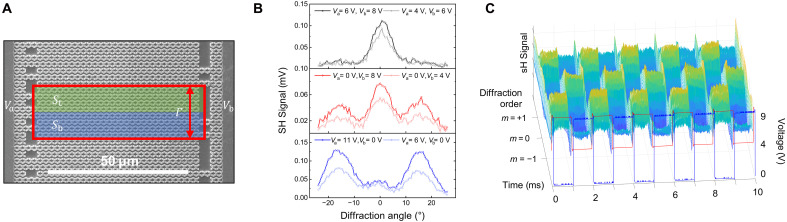
SH beam modulation using phase and amplitude gratings. (**A**) SEM image of fabricated SH phase-grating sample. The green and blue color indicate M_a_ and M_b_ meta-atoms, and the electrodes on the left and right side divided by red dashed line are connected to *V*_a_ and *V*_b_. The top and bottom subcells in a single grating period are denoted as *S*_t_ and *S*_b_. (**B**) Experimental measurement result of the far-field profiles of the SHG intensity for different bias conditions. With the bias configuration shown in (A), applying only *V*_b_ results in an amplitude grating with a 50% duty cycle (bottom), applying only *V*_a_ creates a phase grating with a 50% duty cycle (middle), and applying appropriate *V*_a_ and *V*_b_ can produce a zeroth-order diffraction SH signal only (top). (**C**) Dynamic SH beam diffraction modulation result in time domain. *x* axis indicates time, *y* axis indicates beam diffraction pattern, and *z* axis indicates SH signal. The red curve indicates *V*_a_ (low: 4 V, high: 9 V), and the blue curve indicates *V*_b_ (low: 0 V, high: 9 V).

For the application using local phase tuning of SHG signals, we fabricated electrically induced phase or amplitude grating metasurfaces using two bias voltages, *V*_a_ and *V*_b_. The metasurface consists of four unit structures in the *y* direction, forming a supercell grating period ( Γ = 4 $P_{y}$ = 18 μm), as shown in Fig. 6A. The *M*_b_ meta-atoms in the top subcell (*S*_t_) and the *M*_a_ meta-atoms in the bottom subcell (*S*_b_) are connected to the bias contact *V*_a_ to create a π-phase difference between subcells. In addition, the *M*_b_ meta-atom in *S*_b_ is connected to the bias contact *V*_b_, enabling amplitude grating or fine phase tuning between subcells. Figure 6B shows the measurement results of SH beam diffraction for three different bias sets (*V*_a_ and *V*_b_) with normal incidence of the input pump beam at a wavelength of 10.5 μm. When only *V*_a_ is applied, an SH signal is generated as the phase difference between the meta-atoms within the unit structure deviates from π. In addition, because of the electrode connection configuration, there is always a phase difference of π between *S*_t_ and *S*_b_, resulting in the observation of only the ± first-order diffraction signal (Fig. 6B, bottom). When only *V*_b_ is applied, no SH signal is generated in *S*_t_, and the SH signal appears only in *S*_b_, leading to the induction of the square-wave amplitude grating (*32*). As the voltage *V*_b_ is increased, the ratio between the zeroth and first-order diffraction signals remains constant, while their magnitude increases (Fig. 6B, middle). By appropriately adjusting the bias set (*V*_a_ and *V*_b_), it is also possible to induce the phases of *S*_t_ and *S*_b_ to align, resulting in only the zeroth-order diffraction signal (Fig. 6B, top). Other possible bias configurations and the corresponding SH beam diffraction results are shown in text S5. The electrically induced phase- and amplitude-grating metasurface can be easily modulated by the bias conditions, and we also measured the dynamic modulation of the SH signal in the time domain from the two stages. In Fig. 6C, by applying two synchronized pulses of the bias set [*V*_a_(*t*), *V*_b_(*t*)] to the metasurface appropriately, the zeroth- and first-order SH beam diffraction patterns alternately appear in time.

## DISCUSSION

In this work, we presented and demonstrated a method for electrically controlling the complex amplitude of the effective second-order nonlinear susceptibility in the intersubband polaritonic metasurface for SHG. Using a two meta-atom unit structure with a π phase difference, we demonstrated broadband, complete on/off modulation of the SH signal. Through nonlinear optical interferometric measurements, we showed access to every phase point of SH signal across 360° with a radius of up to 30 nm V^−1^. In addition, we demonstrated SH signal beam diffraction tuning and on/off modulation using amplitude- and phase-grating metasurfaces controlled by local electrical tuning of amplitude and phase. The approach outlined in this paper, which involves the effective IST control and the use of a dual meta-atom structure to modulate the effective nonlinear optical susceptibility, has wide applicability for metasurface devices. We believe that this approach can be extended to most of the mid–infrared (IR) and near-IR regions by using heterostructures with appropriate conduction band offsets. Integrating our electrically tunable metasurfaces with read-in integrated circuits enable two-dimensional phased-array metasurfaces with independent control over each meta-atom’s amplitude and phase. This would allow real-time, programmable wavefront shaping; adaptive focusing; and beam steering, making the metasurface a versatile tool for dynamic holography and optical information processing. Such an architecture could advance applications in quantum photonics and secure communication, offering a scalable, high-resolution, and compact solution for real-time nonlinear optical control.

## MATERIALS AND METHODS

### Numerical simulation

The IST energies and dipole matrix elements induced in the conduction band edge of coupled three quantum wells under a bias electric field were computed using a self-consistent Poisson-Schrodinger solver (Nextnano). The calculated IST energies, dipole matrix elements and measured linewidth, and doping density were used to calculate optical parameters including the permittivities and second-order susceptibility. FDTD simulator (lumerical FDTD) was used for the optimization of the meta-atom design. The out-of-plane and in-plane dielectric constants of the MQW layer [ $\varepsilon_{⊥}\left(\omega \right)$ and $\varepsilon_{∥}\left(\omega \right)$ ] were modeled using the intersubband absorption measurement data and the calculated transition dipole matrix elements (see text S3), and these values were incorporated into FDTD simulations. The simulation duration was set to 3000 fs, and the temperature was assumed to be 300 K. Periodic boundary conditions were applied along the *x* and *y* axes, while a perfectly matched layer boundary condition was used along the *z* axis.

### Device fabrication

The 500-nm-thick MQW layer epitaxially grown on an InP substrate was transferred to the Si substrate by thermo-compression wafer bonding process after the deposition of 20-nm Cr, 50-nm Pt, and 150-nm Au by electron-beam evaporation on the MQW and Si wafer as a bonding layer and a bottom metal plane. The InP substrate on the MQW layer was removed by mechanical polishing and selective chemical etching with 300-nm In_0.53_Ga_0.47_As and 100-nm InP etch-stop layers. Top metal layers of 6-nm Cr and 60-nm Au were deposited by electron-beam evaporator, and 450-nm Si*_x_*N*_y_* dry etch mask layer was deposited by plasma-enhanced chemical vapor deposition. The meta-atom patterns were processed by electron-beam lithography and inductively coupled plasma (ICP) dry etching, and the Si*_x_*N*_y_* mask layer was removed by a buffered oxide etchant. For active device fabrication, mesa structures (400 μm by 400 μm) were patterned by photolithography and dry-etched with ICP etching after the deposition of 450-nm Si*_x_*N*_y_* dry etch mask layer to reduce the current leakage. For the passivation layer, 350-nm Si*_x_*N*_y_* layer was deposited, and the patterned areas were opened by a buffered oxide etchant after photolithography. Top contact metal of 20-nm Cr, 300-nm Au, 20-nm Cr, and 50-nm Au were deposited on photolithography pattern and removed metal layers except contact pads by lift-off process with acetone. The fabricated device was fixed on a copper heat sink by a silver paste. The detailed illustration of the device fabrication process is shown in fig. S3.

### Optical characterization

The reflection spectra of the fabricated samples were measured by FTIR spectrometer equipped with an IR microscope (Bruker, VERTEX70 and HYPERION 1000). For nonlinear optical measurement, a broadly wavelength-tunable quantum cascade laser (QCL) operating in pulse mode (Daylight Solutions Inc., MIRCAT system, tuning range: 909 to 1230 cm^−1^, peak power: 400 mW, repetition rate: 100 kHz, duty cycle: 10%) and a calibrated InSb photodetector [Electro-Optical System Inc., bandwidth (d.c.): 200 kHz] were used. The linearly polarized input beam from the QCL passes through the long pass (LP) filter (cutoff wavelength: 7 μm) and focuses on the metasurface by the ZnSe aspheric lens (numerical aperture: 0.67). The generated SH signal was directed to the detector via the LP filter, the linear polarizer, and the SP filter (cutoff wavelength: 6 μm). The focal spot diameter at the sample position was 2*w* = 48 μm, confirmed by the knife-edge measurement. A Gaussian power profile was assumed for both the FF input pump beam [ $I_{\text{FF}}\left(r\right)=I_{\text{FF}}e^{-2r^{2}/w^{2}},I_{\text{FF}}=\frac{2P_{\text{FF}}}{\pi w^{2}}$ ] and the SH beam [ $I_{\text{SH}}\left(r\right)=I_{\text{SH}}e^{-4r^{2}/w^{2}},I_{\text{SH}}=\frac{4P_{\text{SH}}}{\pi w^{2}}$ ]. The average pump power was measured by a thermal power meter (Thorlabs, S302C). Two different DC bias voltages were applied using two source meters (Keithley SMU 2450, Agilent E3631A). For the dynamic SH signal modulation, two square voltage pulses of 1 kHz frequency were applied using a high-voltage pulse generator (HP 8114A) and a waveform generator (Keysight EDU33212A) connected with a dc power supply (Keysight E36313A) in series. Two square voltage pulses were synchronized by an external trigger connection, and the detector signal was monitored using an oscilloscope (Tektronix TDS2024C). For the SH beam diffraction measurement, the detector with a rectangular aperture on a moving stage scanned zeroth- and first-order diffraction signal with 0.1-mm steps in the lateral direction. For the SH interferogram measurement, the moving stage scanned with 0.5-mm steps, and the interfered signal was observed using the detector with a pinhole (200 μm by 200 μm). Detailed measurement setup is described in fig. S6.

## Supplementary Materials

### This PDF file includes:

Supplementary Texts S1 to S5 Figs. S1 to S9
