# Supplementary material for: Full complex amplitude control of second-harmonic generation via electrically tunable intersubband polaritonic metasurfaces
Source: Sci Adv. 2025 Jul 25;11(30):eadw8852. doi: 10.1126/sciadv.adw8852 (PMC12292913; doi:10.1126/sciadv.adw8852)
Supplement: Supplementary file 1 — Supplementary Texts S1 to S5 Figs. S1 to S9 [file sciadv.adw8852_sm.pdf]

Science Advances
AAAS

Supplementary Materials for

Full complex amplitude control of second-harmonic generation via electrically tunable intersubband polaritonic metasurfaces

Jaeyeon Yu *et al.*

Corresponding author: Jongwon Lee, jongwonlee@unist.ac.kr

Sci. Adv. **11**, eadw8852 (2025)
DOI: 10.1126/sciadv.adw8852

This PDF file includes:

Supplementary Texts S1 to S5
Figs. S1 to S9

1. Chords through the cusp from second order susceptibility

In the MQWs which have eigenstates with equally spaced energy differences ($\omega_{31}=2\omega_{21}$), the second-order nonlinearity from the MQWs mainly induced by double resonant transitions between intersubband states, and the second-order susceptibility can be simplified as follows.

$$\chi_{zzz}^{(2)} \approx \frac{N_e e^3}{\hbar^2 \varepsilon_0} \frac{z_{12} z_{23} z_{13}}{(\omega_{31}-2\omega-i\gamma_{31})(\omega_{21}-\omega-i\gamma_{21})} \approx \frac{N_e e^3}{2\hbar^2 \varepsilon_0} \frac{z_{12} z_{23} z_{13}}{\left[\omega_{21}-\omega-i\left(\frac{\gamma_{21}}{2}+\frac{\gamma_{31}}{4}\right)\right]^2+\left(\frac{\gamma_{21}}{2}-\frac{\gamma_{31}}{4}\right)^2}, \quad \text{(S1)}$$

where N_e is the charge density, e is the electron charge, ε_0 is the vacuum permittivity, $\hbar$ is the reduced Planck's constant, and z_{ij} are the dipole moment elements between levels i and j. To maximize phase change as a function of applied vias voltage, we designed the initial values of the IST energies (E_{12} and E_{23}) in MQWs without applied voltage to be equal, and the changes in these energies with applied voltage to be the same. Expanding Eq. S1 using a Laurent series, and considering the measured values of transition linewidths $2\gamma_{12}\approx\gamma_{13}$ (see Supporting Materials Section 3), higher-order terms that rapidly vanish as IST energies shift away from the target wavelength with applied voltage can be neglected, resulting in the simplified equation:

$$\chi_{zzz}^{(2)}(V) \approx \frac{N_e e^3 z_{12} z_{23} z_{13}}{2\hbar^2 \varepsilon_0} \frac{1}{\left[\omega_{21}(\mathrm{V})-\omega-i\left(\frac{\gamma_{21}}{2}+\frac{\gamma_{31}}{4}\right)\right]^2}, \quad \text{(S2)}$$

where $\omega_{21}(\mathrm{V})$ is the resonant frequency for transition between states 2 and 1. When represented as a polar function in the complex plane, this equation can be transformed into the following:

$$Re\left(\chi_{zzz}^{(2)}\right) = -2f \cdot \sin(\theta) \cdot (\sin(\theta)+1) \quad \text{(S3)}$$

$$Im\left(\chi_{zzz}^{(2)}\right) = 2f \cdot \cos(\theta) \cdot (\sin(\theta)+1) \quad \text{(S4)}$$

where $f = \frac{z_{12} z_{23} z_{13} N_e e^3}{8\hbar^2 \varepsilon_0 \Gamma^2}$, $\theta = tan^{-1}\left(\frac{\Gamma^2-(\omega_{21}(V)-\omega_{21}(0))^2}{2\Gamma(\omega_{21}(V)-\omega_{21}(0))}\right)$ and $\Gamma = \hbar\left(\frac{\gamma_{21}}{2}+\frac{\gamma_{31}}{4}\right)$. These Eq. S3 and S4 represent a cardioid function, denoted as $r(\theta) = 2f \cdot (1-\cos(\theta))$. The cardioid function exhibits unique properties, including the feature of "chords through the cusp", where points $P = r(\theta)$ and $Q = r(\theta+\pi)$ lie on a chord passing through the cusp (origin). These chords all have equal lengths [Ref. 28]. Figure 1a in the main manuscript shows a blue graph representing $r(\theta) = \frac{1}{2} \cdot (1-\cos(\theta))$, and a red graph representing $r'(\theta') = \frac{1}{2} \cdot (1-\cos(\theta'-\pi))$, where the two graphs exhibit a π-phase difference. Figure 1B in the main manuscript displays the sum of these two functions at the same phase ($\theta = \theta'$), which results in a constant value of 1 ($r(\theta)+r'(\theta) = 1$). Using this approach, if the two second-order susceptibilities originating from the same MQWs have a π-phase difference, their maximum sum will be a constant value $4f$, independent of the polar angle. Moreover, when $\theta' \neq \theta$, the interference between the two functions causes the maximum radius $4f$ to form an outer boundary, with every point inside the circle represented by the two variables θ' and θ.

2. Comparison of multiple quantum wells structures

Figure S1 compares the changes in phase and amplitude of MQW structures for nonlinear polaritonic metasurfaces. The first experimental demonstration of nonlinear polaritonic metasurfaces is based on coupled double quantum wells, as shown in Fig. S1A [Ref. 21]. Figure S1D shows the voltage-dependent changes in IST energies. At zero bias, E_{12} and E_{13} are 153 meV and 307 meV, respectively, which induce high second-order susceptibility. However, the slopes of E_{12} and E_{13} with respect to the applied voltage are 0.285 meV cm V^{-1} and 0.036 meV cm kV^{-1}, respectively. The magnitude and phase changes in second-order susceptibility at a central wavelength of 8.1 μm are presented in Fig. S1G, and for comparison, the voltage range is from -40 kV cm^{-1} to 40 kV cm^{-1}. The maximum-to-minimum intensity ratio is 1.78, and the maximum phase difference is 105°. Although the QCSE introduces some variation in coupled double quantum wells, the changes are relatively small.

To further increase the modulation in magnitude and phase of second-order susceptibility with voltage, the slope of E_{13} needed to be enhanced. In Ref. S3, this was achieved by employing a coupled three quantum wells structure, as shown in Fig. S1B. The change in the energy levels of electron subbands under applied z-directional electric field F_z can be calculated using first-order perturbation theory as $E_n^1 = eF_z\langle\psi_n^0|z|\psi_n^0\rangle = eF_z z_{nn}^0$, and the change of IST energy difference between the n and m states can be determined as $\Delta E_{nm} = eF_z(z_{mm}^0 - z_{nn}^0) + O(F_z^2)$. For spatially separated electron subbands, the first-order energy correction term becomes dominant and varies linearly with the applied voltage. In the case of three coupled quantum wells, it is easier to adjust the positions of the three electron subbands. Figure S1E shows the voltage-dependent changes in E_{12}, E_{23}, and E_{13}. At zero bias, E_{12} and E_{13} are 126 meV and 260 meV, respectively, and their slopes with respect to voltage are 0.451 meV cm V^{-1} and 0.386 meV cm V^{-1}, respectively. The magnitude and phase variations of the second-order susceptibility at a central wavelength of 10 μm are presented in Fig. S1H, with a voltage range of -40 kV cm^{-1} to 40 kV cm^{-1}. The maximum-to-minimum intensity ratio is 4.04, and the phase shift is 192°. However, the change in E_{13} is still less than twice that of E_{12}.

To maximize the phase change as a function of the applied bias voltage, we designed the initial values of IST energies E_{12} and E_{23} to be equal in MQWs without applied voltage, and the voltage-dependent changes in these energies were aligned. In the three coupled quantum wells in this work, Fig. S1C, at zero bias, E_{12} and E_{13} are 123 meV and 246 meV, respectively, and their slopes with respect to voltage are 0.336 meV cm V^{-1} and 0.672 meV cm V^{-1}, respectively, and the conditions of cardioid function are satisfied. Figure S1I shows the magnitude and phase changes of the second-order susceptibility at a central wavelength of 10 μm. For the voltage range from -40 kV cm^{-1} to 40 kV cm^{-1}, the maximum-to-minimum intensity ratio is 3.92, and the phase shift is 223°.

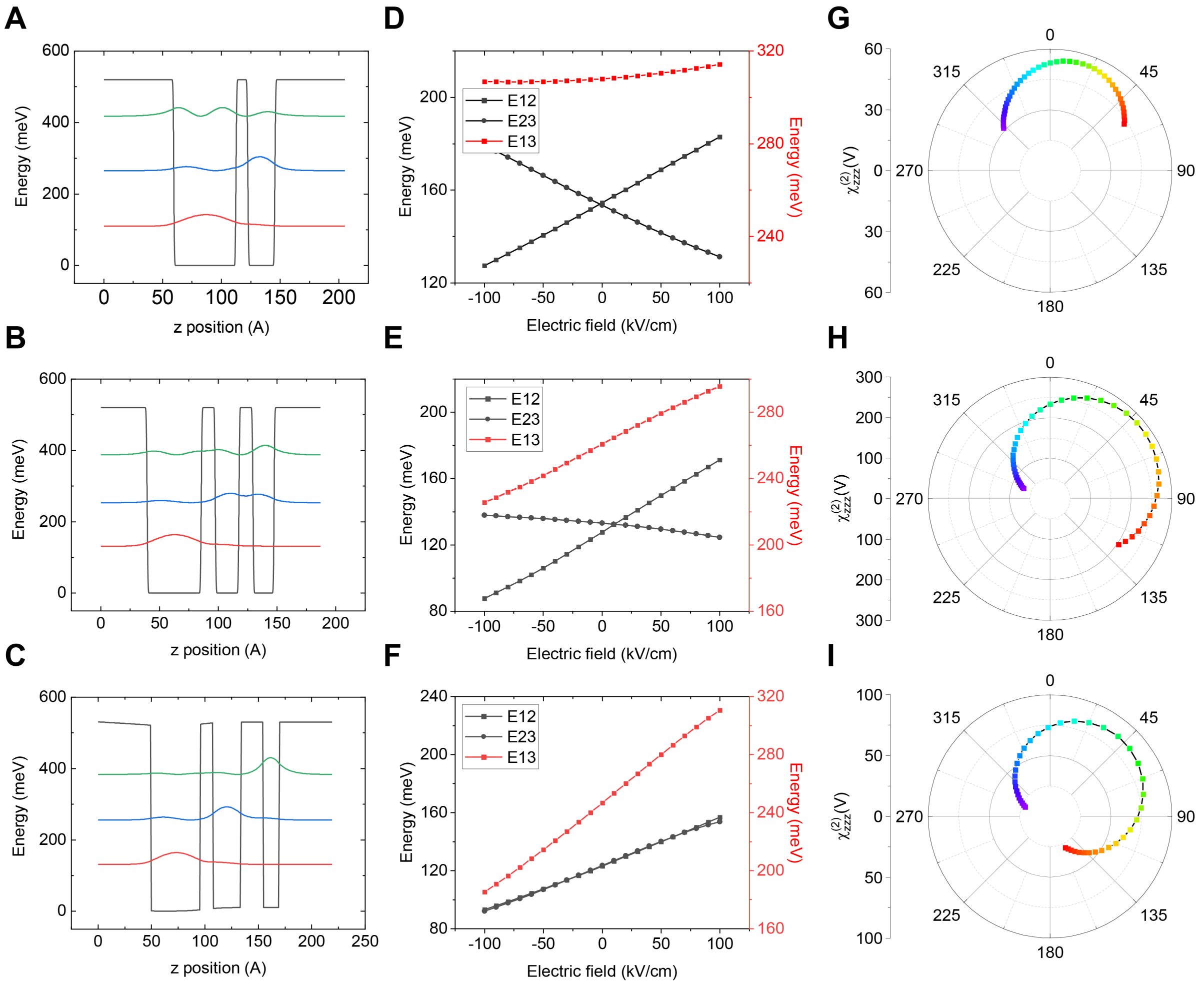

Fig. S1. **Comparison of optical properties of MQWs**. Conduction band edge of MQWs, IST energy changes as a function of applied static electric field and the voltage-dependent changes in second order susceptibility of [Ref. 21] (**A, D, G**), [Ref. 11] (**B, E, H**), and this work (**C, F, I**).

3. Intersubband absorption measurement

The experimental setup for intersubband absorption measurement was reported in Ref. S3. Figure S2A shows experimentally measured absorbance spectrum of the MQW sample which is sandwiched between optically thick gold layers. Because the MQW absorbs only TM polarized light along the growth direction, the intersubband absorption coefficient can be determined by normalizing the TM polarized signal to TE polarized signal after passing the MQW. The intersubband absorption coefficient, α_W, is expressed as [Ref. 23]:

$$\alpha_W = -\frac{1}{L_{int}} \ln(10) \log_{10}\left(\frac{I_{TM}}{I_{TE}}\right) \qquad \text{(S5)}$$

where L_{int} is the MQW interaction length of the multipath sample piece, I_{TM} and I_{TE} are the measured signal for TM and TE polarization, respectively. Transition energies of $\hbar\omega_{12} = 122$ meV, $\hbar\omega_{13} = 257$ meV and $\hbar\omega_{14} = 357$ meV were obtained which are corresponding to E_{12}, E_{23}, and E_{13}, respectively. The transition linewidths are $2\hbar\gamma_{12} = 16.4$ meV, $2\hbar\gamma_{13} = 32$ meV and $2\hbar\gamma_{14} = 60$ meV, and the intersubband absorption coefficient, α_W, are 8.92×10^3 cm^{-1}, 6.09×10^2 cm^{-1} and 1.29×10^3 cm^{-1} for the 1-2, 1-3 and 1-4 transitions, respectively. From the intersubband absorption measurement, the imaginary part of the surface normal component of dielectric function can be determined using the equation expressed as a function of the absorption coefficient as:

$$Im(\sqrt{\varepsilon_\perp}) = \frac{\lambda}{4\pi}\alpha_W \qquad \text{(S6)}$$

From the equation, we extracted the imaginary part of $\varepsilon_\perp$ using the absorption coefficient spectra from the intersubband absorption measurement. In a different way, the surface normal component of dielectric function $\varepsilon_\perp$ of the MQW structure can be modeled as following equation:

$$\varepsilon_\perp(\omega) \approx \varepsilon_{core}(\omega) + \frac{e^2 N_e}{\varepsilon_0 \hbar}\left[\frac{z_{12}^2}{(\omega_{12}-\omega)-i\gamma_{12}} + \frac{z_{13}^2}{(\omega_{13}-\omega)-i\gamma_{13}} + \frac{z_{14}^2}{(\omega_{14}-\omega)-i\gamma_{14}}\right] \qquad \text{(S7)}$$

where ε_{core} is the averaged dielectric constant of the undoped semiconductor heterostructures, N_e is the averaged doping density, *e* is the electron charge, *ω* is the pump frequency, $\hbar\omega_{ij}$, $\hbar\gamma_{ij}$, and ez_{ij} are the transition energy, linewidth, and dipole moment, respectively, for the intersubband transitions between the electron subband *i* and *j*. We estimated the averaged electron density, N_e, to calculate the second-order nonlinear susceptibility and the averaged dielectric constant to determine MQW dielectric constant in surface parallel direction which is expressed as:

$$\varepsilon_\parallel(\omega) \approx \varepsilon_{core}(\omega) + i\frac{N_e e^2 \tau_D}{\varepsilon_0 \omega m^*(1-i\omega\tau_D)} \qquad \text{(S8)}$$

where $\tau_D \approx 10^{-13}$ s is the Drude relaxation time and we assumed free electron motion in the plane of the semiconductor layer. Figure S2B shows fitted graph absorbance spectrum based on the model.

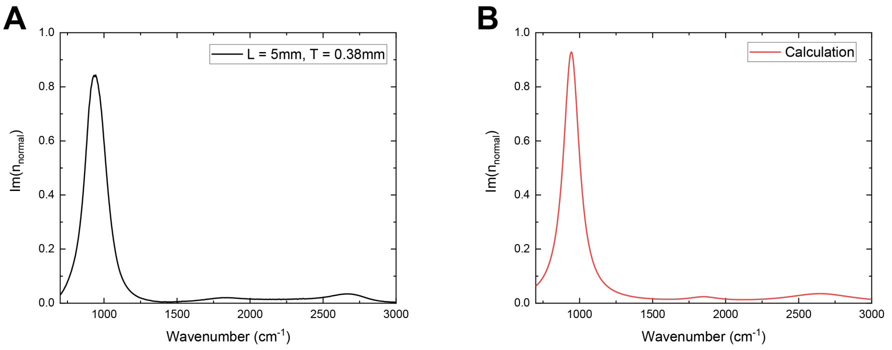

Fig. S2. **Intersubband absorption characterization**. (**A**) Measured intersubband absorption spectrum by normalizing the TM signal to the TE signal passing through MQW after baseline subtraction processing. (**B**) Model calculated intersubband absorption spectrum using the experimentally measured parameters.

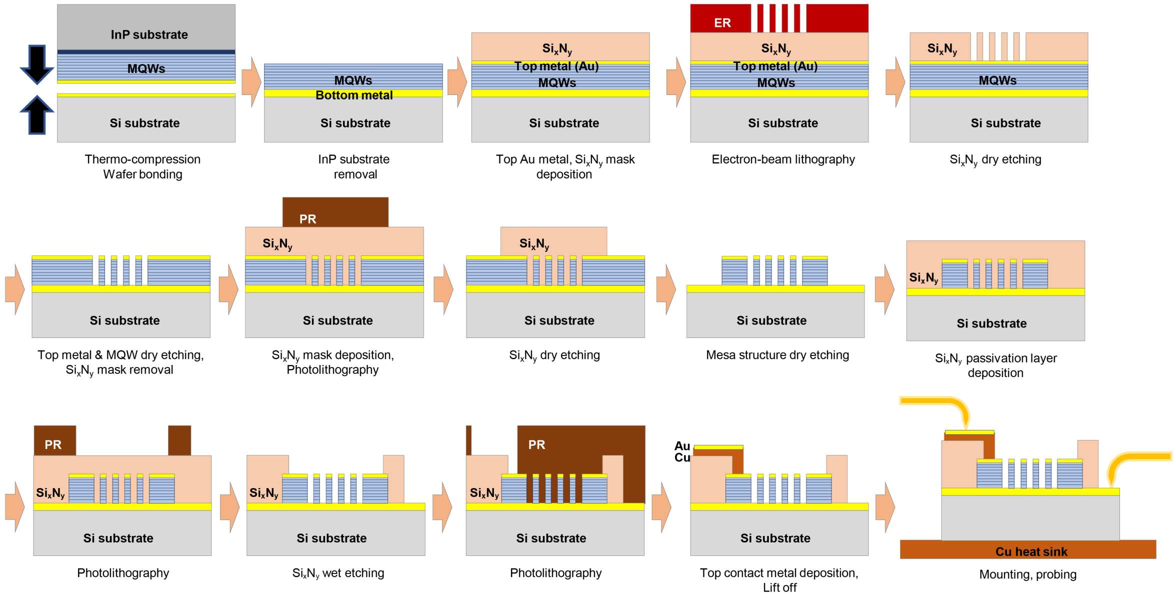

Fig. S3. Metasurface fabrication processes.

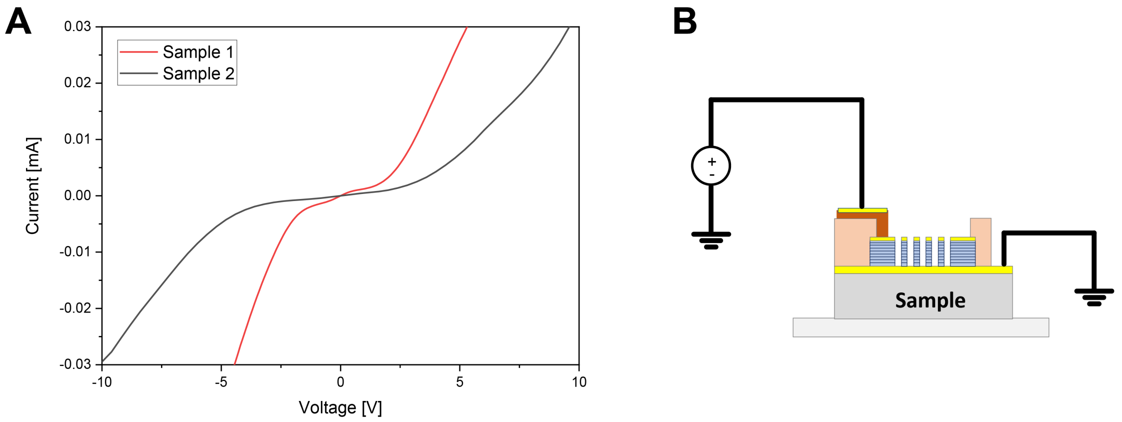

Fig. S4. Current-voltage characterization. (**A**) Current-voltage characteristic of the fabricated metasurfaces. The electrode of Sample 1 is connected to the full metasurface of M_a meta-atoms only, and the electrode of Sample 2 is connected to only half of the metasurface for the separation of M_a and M_b meta-atoms. (**B**) Schematic of the current-voltage characteristic measurement setup.

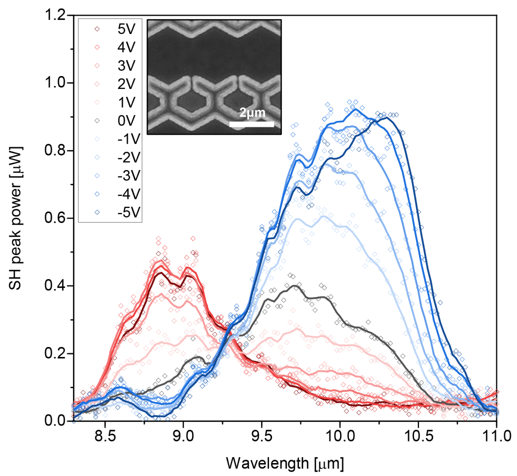

Fig. S5. Additional nonlinear characterization. Measured SH signal spectra (dot - measured data, line - moving average) of only a half-side meta-atom structured metasurface (inset) as a function of the input pump wavelength for different bias voltages from -5 V to 5 V.

4. SH interferometer measurement

Although the differences in phase and amplitude of the SH signal from diffraction pattern can be roughly estimated as a function of the applied voltage, a more precise measurement was achieved by designing the SH interferometer as shown in Fig. S6B. An input pump beam from the QCL was incident on both the sample and the SHG reference via a 7:3 (transmission:reflection) beam splitter (BS). The SHG reference employed was a nonlinear metasurface used in a previous study [Ref. 21]. The SHG reference and ZnSe lens 2 were mounted on a linear actuator (moving stage 1), which moves along the optical axis to adjust the phase. To ensure precise movement along the optical axis, a long-pass (LP) filter, which reflects visible light, was utilized, allowing the focal spot to remain fixed while the actuator moved. The position of the focal spot was monitored via a CCD camera to ensure accuracy during measurements.

Figure S7 shows the SH signal measured as the position of the detector, equipped with a 200 × 200 mm^2 pinhole and mounted on moving stage 2, was shifted perpendicularly to the optical axis, recording the interference pattern between the SH signal generated by the sample and that generated by the SHG reference. To find the initial position of the SHG reference, the position of moving stage 1 was adjusted to produce a specific interfered pattern between the SH signal from the sample and the SHG reference, as shown in Fig. S7B. When the SHG reference is in its initial position, the symmetric pattern corresponding to destructive interference at the center point, as shown in Fig. S7B, can be observed. When no voltage is applied, and thus no SH signal is generated from the sample, only the SH signal from the SHG reference is observed as Fig. S7A. If the SHG reference is not at its initial position, even when voltage is applied to the sample, the interference pattern becomes asymmetric or entirely different, as seen in Fig. S7C and S7D. After confirming the initial position, the desired voltage was applied to the sample, and the SH interferogram was measured. Measurements were repeated 10 times, and the phase was extracted by fitting the resulting data to a sinusoidal waveform. Figure S8 shows the measured interferogram for specific voltage combinations, including six points with an intensity of 60 nm V^{-1} and phase differences of 45°(#1), 90°(#2), 135°(#3), 225°(#4), 270°(#5), and 315°(#6), as well as eight points with an intensity of 30 nm V^{-1} and phase differences of 0°(#7), 45°(#8), 90°(#9), 130°(#10), 180°(#11), 225°(#12), 270°(#13), and 315°(#14). It is noted that the motorized actuator used for the moving state of the SHG reference has a spatial resolution of 0.1 μm. However, there was a limitation in acquiring a sufficient number of interference data points required to extract more SHG signal phase points. Furthermore, due to the intrinsically low intensity of the SHG signal, there were constraints in applying techniques such as lateral beam expansion commonly used in linear optics.

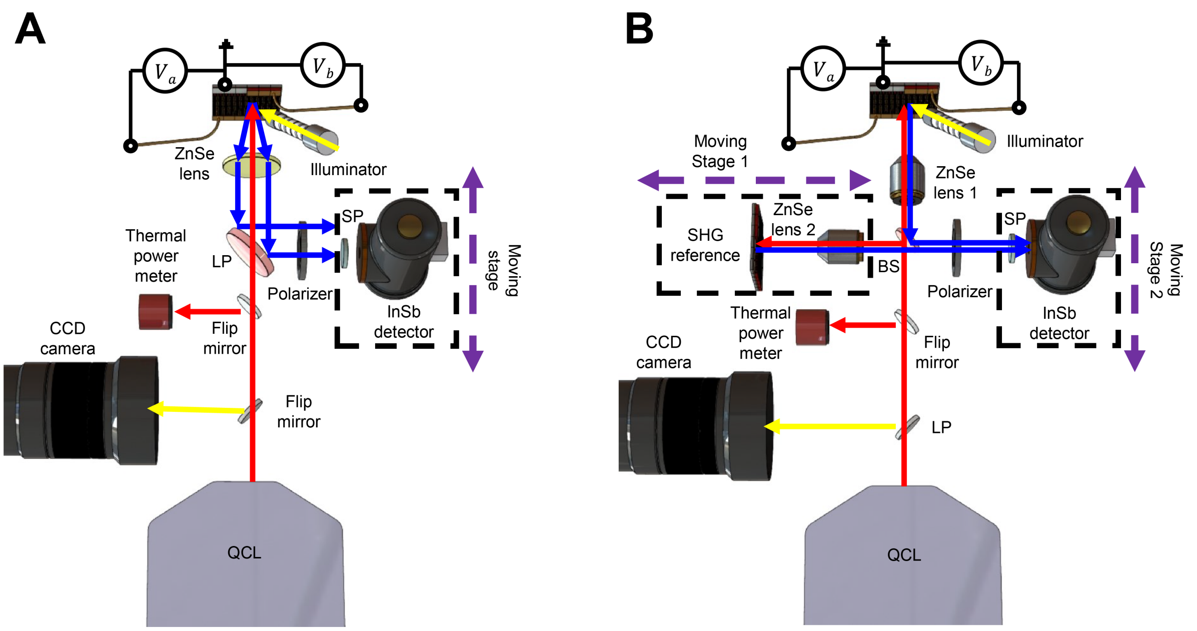

Fig. S6. **Experimental setups**. (**A**) Nonlinear optical measurement setup. (**B**) SH interferometer setup. The red arrow represents the QCL input, the blue arrow indicates the SH output, and the yellow arrow shows the visible light used for alignment. The components within the black dashed boxes are mounted on the translation stages, which move along the direction indicated by the purple dashed arrow.

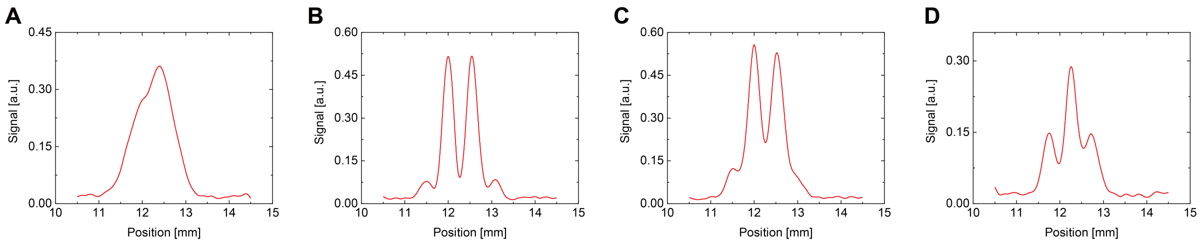

Fig. S7. **Transverse SH intensity profile measured by shifting the position of the detector, equipped with a pinhole and mounted on moving stage**. SH interference patterns measured with applied voltage of (**A**) V_a : 0 V, V_b : 0 V, with applied voltage of V_a : 6 V, V_b : 0 V at (**B**) the initial position and (**C, D**) when the SHG reference is out of the initial position.

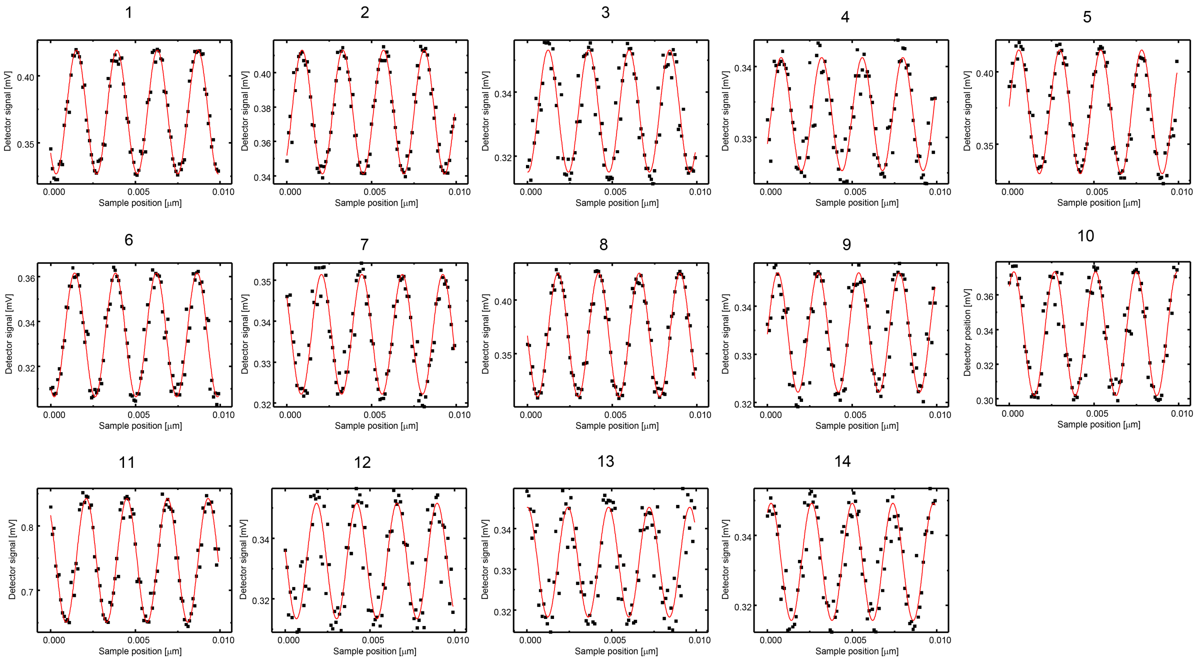

Fig. S8. **Measured SH interferograms at the pump wavelength of 9.8 μm under the voltage combinations V_a and V_b.** #1 (2.78V, -5.14V), #2 (6.12V, -2.51V), #3 (6.32V, 0.14V), #4 (-4.65V, 2.24V), #5 (-1.89V, 5.28V), #6 (0.25V, 6.50V), #7 (0.83V, 6.67V), #8 (-2.08V, -4.17V), #9 (0V, -2.50V), #10 (2.92V, -0.83V), #11 (6.67V, 0.83V), #12 (-3.75V, -2.08V), #13 (-2.50V, 0V), #14 (-0.83V, 2.92V).

5. SHG amplitude and phase grating measurement

To fully demonstrate all possible combinations of amplitude and phase in grating metasurfaces, four electrode contacts are required. However, there were technical limitations in fabricating more than two electrode contact pads. As illustrated in Fig. S9a, five possible voltage configurations for the SH amplitude and phase grating metasurface were investigated. The first configuration is the case that only single side of meta-atoms in two subcells are connected to V_a and V_b separately. In this case, each voltage shows same amplitude grating operation, and the grating becomes in-phase and shows strong zeroth signal when the voltages are applied simultaneously. The second configuration is similar to the first case, but the opposite sides of meta-atoms are connected to V_a and V_b separately. In this case, each voltage shows same amplitude grating operation with opposite phase profile, so the grating becomes p-phase grating when the voltages are applied simultaneously. The third configuration is the case that the whole single side of meta-atoms are connected to V_a, and opposite side of meta-atoms in half subcells are connected to V_b. In this case, the grating becomes in-phase applying V_a and operates amplitude grating applying V_b. When V_a and V_b are applied properly, $\pi/2$-phase grating operation can be obtained which shows same diffraction pattern compared to amplitude grating but with a different principle. The fourth configuration is the case that the different side of meta-atoms for each subcells are connected to V_a, and single side of meta-atoms in half subcells are connected to V_b. In this case, π-phase grating operation can be obtained when V_a is applied, and V_b shows amplitude grating operation which is same as previous cases. When V_a and V_b are applied properly, the grating becomes in-phase due to amplitude and phase matching between subcells. The fifth configuration is the case that the different side of meta-atoms for each subcells are connected to V_a, and remained opposite sides are connected to V_b. In this case, each voltage shows same π-phase grating operation with opposite phase profile, and the grating becomes out of phase and shows canceled signal when the voltages are applied simultaneously.

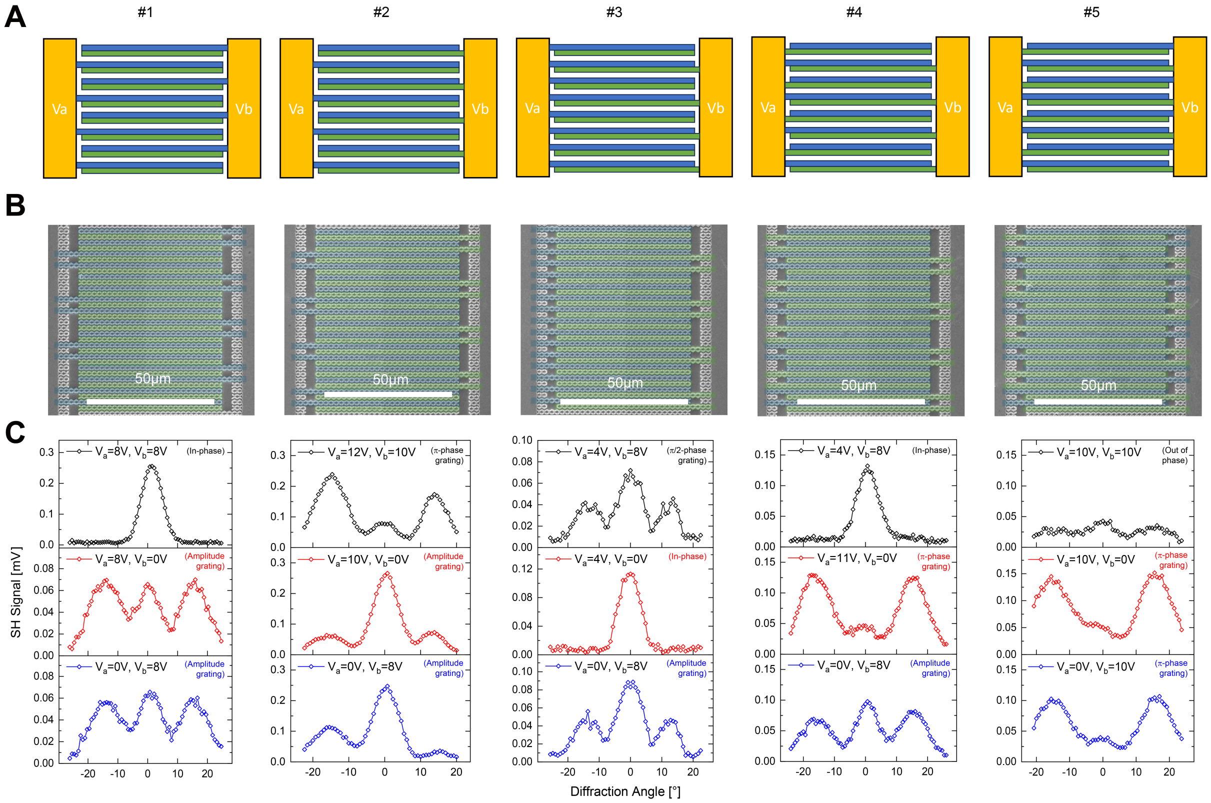

Fig. S9. **SH beam modulation for various bias contact configurations**. (**A**) Five different voltage bias configurations for SH phase or amplitude grating metasurfaces. (**B**) SEM image of each sample corresponding to (**A**). (**C**) SHG beam diffraction measurement results for three different bias sets. The red graph indicates the operation when only V_a is applied, and the blue graph indicates the operation when only V_b is applied. The black graph is the representative case when V_a and V_b are applied properly.
